# A Case of Restless Arm Syndrome and the Effective Use of Gabapentin

**DOI:** 10.1155/crnm/1198600

**Published:** 2026-02-03

**Authors:** Jasmine Lim Shimin

**Affiliations:** ^1^ Internal Medicine, Geriatrics, Ng Teng Fong General Hospital, National University Healthcare Systems (NUHS), 1 Jurong East Street 21, 609606, Singapore

## Abstract

Restless arm syndrome (RAS) is an uncommon neurological condition that is characterized by an irresistible urge to move the upper limbs, often worse at rest and transiently relieved by movement. RAS is an upper limb variant of restless legs syndrome (RLS), a more common neurological condition, also known as Willis–Ekbom disease. In view of its atypical presentation, RAS is often underdiagnosed. RAS is reported to share the same risk factors as RLS with its prevalence increasing with age, in the presence of certain conditions, and associated with the use of medications like antipsychotics and antidepressants. A case of possible RAS is described here in a patient who had experienced a few years of RLS‐like symptoms and had been on various antidepressants for his depression. His symptoms markedly improved with a trial of gabapentin, which subsequently raised the possibility of RAS as a diagnosis. Clinicians should consider RAS as a differential in patients who experience symptoms suggestive of RLS in the presence of associated risk factors.

## 1. Introduction

Restless legs syndrome (RLS) has been documented in the literature to affect not only the lower limbs but also other parts of the body such as the arms, abdomen, pelvic area, and face. This deviation from its usual clinical presentation is also termed the RLS variant, with the restless arm variant being the most common [1]. The prevalence of RLS increases with age and has been reported in up to 35% of older adults [2]. The prevalence of restless arm syndrome (RAS), on the other hand, is largely unknown.

RAS has been found to be clinically similar to RLS except for its symptom localization. RAS can be clinically defined with reference to the diagnostic criteria for RLS developed by the International Restless Legs Syndrome Study Group (IRLSSG). It is characterized by (1) an urge to move the limb with or without an unpleasant sensation; (2) is enhanced with rest/inactivity; (3) happens more in the night; (4) is partially or totally relieved with movement and is not accounted for by another medical or behavioral condition. [3]. A proportion of patients experience debilitating complications including significant sleep disturbances and depression which further impact quality of life. RLS is also recognized as one of the causes of primary sleep disorders in older adults [4, 5]. While dopaminergic agonists have traditionally been used as first‐line treatment in RLS, gabapentinoids are increasingly recognized as an effective alternative [6–8].

While RLS may be more commonly encountered in the clinical setting, RAS is often underrecognized. A possible diagnosis of RAS in this case was made in retrospect following unsuccessful interventions for his insomnia. It also serves to further highlight the complexity of managing sleep disorders in older adults.

## 2. Case Presentation

The patient was a 94‐year‐old Chinese man who was referred to the geriatrics clinic initially for worsening agitation and low mood complicated by poor sleep and weight loss. He had a significant history of ischemic heart disease, Stage 3 chronic kidney disease, and a previous left middle cerebral artery (MCA) stroke complicated by right‐sided hemiparesis and poststroke depression. He had been on sertraline 50 mg every morning for his depression prior to the review. Following his assessment, his antidepressant was switched to mirtazapine to aid with his sleep as well as his appetite. He responded well to mirtazapine, and his dose was gradually increased to 22.5 mg every night.

**Figure 1:**
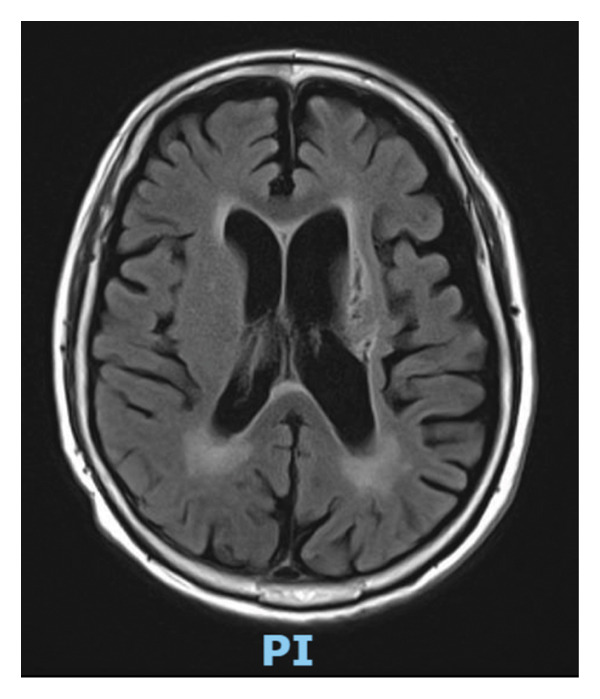
T2‐FLAIR of a magnetic resonance imaging (MRI) scan of the brain with reported old infarcts in the left striatocapsular region, right lentiform nucleus, right hemipons, and right cerebellum.

His family reported intermittent, repetitive movements of his left arm at night, with occasional movements of his left lower limb about 3–4 months later. It was described as a repeated banging of his left arm on the side of his bed. This was associated with nocturnal awakenings and interrupted sleep. There were no similar movements during the day and no report of other limb involvement. On further history, he reported a “need to move it as he is used to it.” He denied any abnormal sensations or any associated pain. Despite his poor sleep at night, there was no excessive daytime sleepiness and no increase in daytime naps. Examination showed increased tone over his right upper and lower limbs with a fixed flexion deformity in his right elbow. The power on his right side was a grade of 1/5. The tone over his left upper and lower limbs was normal, and his power was a grade of 5/5. Magnetic resonance imaging of his brain showed old infarcts in the left striatocapsular region, right lentiform nucleus, right hemipons, and right cerebellum (Figure 1).

Nonpharmacological management for sleep was advised with little observed benefit. Melatonin was added on with a similarly poor response. Benzodiazepines and the nonbenzodiazepine hypnotics were avoided in this frail older adult in view of concerns of their associated side effects. On one of his subsequent reviews, he reported nonspecific cramps in his lower limbs. Examination showed the presence of intact peripheral pulses in both his lower limbs, no new neurological deficits, and no lower limb edema. A decision was made then to trial him on gabapentin 100 mg every night for his cramps in addition to his persistent sleep issues. During his review in clinic 3 months later, his family reported a marked improvement in both his arm movements and his insomnia. His gabapentin was increased to 200 mg every night with further improvement in his symptoms.

In retrospect, a diagnosis of possible RAS was considered. Potential risk factors like family history, iron deficiency, and medications were then explored. He was reported to have no family history of RAS or RLS. While his last iron panel showed the presence of mild iron deficiency, he had been on iron supplementation since. On review of his medication history through the years, he had never been on any antipsychotics prior to his presentation. In view that his mood has been stable and antidepressants may have potentially contributed to his RAS symptoms, his mirtazapine was gradually weaned down and stopped. He was not switched to a dopamine agonist in view of his favorable response to gabapentin and the concerns of possible side effects of dopamine agonists in older adults.

## 3. Discussion

It was found that the clinical picture of RAS does not differ from RLS except for its symptom localization in the arms. A previous literature review of 10 reported RAS cases was noted to fulfill the IRLSSG criteria for RLS. They all reported an urge to move their arms which was accompanied by various abnormal sensations [4]. Supportive features include a family history of RLS, a lack of excessive daytime sleepiness, and a response to dopaminergic agonists [2, 3]. While the patient in this case denied abnormal sensations, there appeared the urge to move his right upper limb, which was more prominent at night while at rest. His lack of daytime sleepiness, evident by the lack of naps in the day, aims to support this diagnosis.

Of 10 cases of RAS reported, symptoms were bilateral in 8 patients and right‐sided in 2 [4]. The patient in this case experienced asymmetric symptoms in his nonparetic arm, with occasional reported movements in his nonparetic leg. A case of poststroke RAS involving the paretic limb had been reported previously [9]. It was noted in another study that unilateral poststroke RLS often happened when lesions were found in the pontine base and tegmentum, while bilateral RLS often occurred when lesions were found in the corona radiata and adjacent basal ganglia [10]. It is insufficient to conclude whether the locality of his strokes contributed to his presentation in this case.

Both RLS and RAS can be classified either as primary or secondary RLS. Primary RLS is idiopathic and is often thought of as a familial condition, although the etiology is largely unknown. Secondary RLS has been related to conditions such as iron deficiency, chronic renal failure, and certain psychiatric medications [4, 11]. Secondary RLS tends to occur later in life, with a more rapid and severe trajectory [12]. Medications implicated in secondary RLS include neuroleptics and antidepressants, which have been found to worsen RLS in various studies. Olanzapine‐induced RAS has previously been described in other reports [13]. The antipsychotics commonly implicated include olanzapine and quetiapine, while antidepressants like mirtazapine have been associated with higher rates of RLS [11]. RAS symptoms appeared in this patient about 3 months following the commencement of mirtazapine but more shortly after an increase in its dose to 15 mg every night.

RLS has been linked to changes in dopamine balance, and successful treatment with dopaminergic agents further suggests a dopamine decline in the brain of patients with RLS [14]. While dopaminergic agonists were previously known as first‐line treatment in RLS, the risk of augmentation (worsening RLS symptoms) with their use has been increasingly recognized. The risk of augmentation increases with the duration and dose of any dopaminergic agents used [6]. Alpha‐2 delta calcium channel ligands (gabapentinoids) can be considered as an alternative initial treatment in some patients. They have been found to be effective and come with a lower risk of augmentation. Patients who may benefit from the use of gabapentinoids instead of dopamine agonists as first‐line treatment include those with significant sleep disturbance, insomnia, or a comorbid pain syndrome [6, 7]. Starting doses of gabapentin can be tried at 300 mg at night (or 100 mg for older adults), although doses found to be efficacious have been reported to be as high as 1200–1800 mg daily [8]. In the case of this patient, a dose of 200 mg at night appears to control his symptoms sufficiently while balancing the risk of side effects at higher doses.

The patient in this case had ongoing symptoms for 3 years before being diagnosed with possible RAS. In view of the atypical presentation of RAS, diagnosis may be delayed in this group of patients. RLS and its variants are important differentials to consider as treatment may potentially improve symptoms and quality of life.

## Funding

No funding was received for this manuscript.

## Ethics Statement

The author has nothing to report.

## Consent

Informed consent for publication of the above clinical details was obtained from the proxy.

## Conflicts of Interest

The author declares no conflicts of interest.

## Data Availability

Data sharing is not applicable to this article as no datasets were generated or analyzed during the current study.
